# Hindrances and Enablers of Healthy Eating Behavior Among College Students in an HBCU: A Qualitative Study

**DOI:** 10.1007/s40615-024-02108-8

**Published:** 2024-07-30

**Authors:** Janet Antwi, Yetunde Olawuyi, Innocent Opara, Modupe Ifafore

**Affiliations:** Department of Agriculture, Nutrition and Human Ecology, Prairie View A&M University, 100 University Dr, Prairie View, TX 77446 USA

**Keywords:** Healthy eating behavior, Enablers, Hindrances, College, HBCU

## Abstract

Research indicates widespread unhealthy eating habits among college students, posing long-term health risks. This study at a Historically Black College and University (HBCU) aimed to explore the perceived obstacles and facilitators to healthy eating among college students, using the social ecological model (SEM). Through focus group discussions and key informant interviews, the study identified several barriers to healthy eating, including challenges in accessing federal food assistance resources, gaps in nutrition knowledge, cost concerns, limited food variety on campus, difficulty accessing grocery stores, and a lack of cooking skills. To address these barriers, participants suggested various solutions, such as implementing cooking demonstrations, providing nutrition education, increasing food variety on campus, offering gardening opportunities, adjusting cafeteria hours for more flexibility, making fresh produce more available on campus, assisting students in accessing federal food assistance programs, and providing transportation to nearby grocery stores. The findings highlight the need for targeted interventions to promote healthier dietary behaviors among college students, particularly those attending HBCUs. By addressing the identified barriers and implementing the suggested solutions, initiatives can be developed to support students in making healthier food choices, ultimately reducing long-term health risks associated with unhealthy eating habits.

## Introduction

Poor nutrition and associated adverse health outcomes are pressing public health issues among college populations in the United States [1]. Studies consistently highlight concerning trends of unhealthy diets, weight gain, disordered eating, and increased chronic disease risk beginning in early adulthood and persisting through higher education [2, 3]. For example, research shows declines in consumption of fruits, vegetables, fiber, and key micronutrients coupled with increased intake of fat, sugar, and overall calories as students transition into college environments [4–6]. These shifts correspond with lifestyle changes like irregular meals, increased snacking and eating out, alcohol consumption, and stress [7]. Consequently, nutrition-related conditions like hypertension, metabolic disorders, cardiovascular diseases, diabetes, and certain cancers emerge in early adulthood and currently burden substantial segments of college populations nationwide [8]. These nutrition issues can also negatively impact academic achievement, workforce readiness upon graduation, and the trajectory of chronic diseases long-term when left unaddressed [9].

College students have a unique demography when it comes to their eating habits and nutrition. They often face numerous challenges and barriers that can hinder their ability to maintain a healthy diet. These challenges can vary from limited time and financial constraints to the prevalence of unhealthy foods on campus [6]. The college years are a critical period for the development of lifelong health behaviors, and understanding the barriers and solutions to healthy eating in this context is crucial for the overall well-being of students. Significant disparities exist across racial/ethnic groups and college settings. Studies consistently report even higher rates of overweight, obesity, diabetes, and metabolic abnormalities among racially minoritized students, including those at minority-serving institutions (MSI) such as HBCUs relative to other universities [10–12]. These nutrition-related health disparities often have roots in early life inequality yet widen further amid college contexts like Hispanic-serving institutions (HSIs) and HBCUs with high enrollment of first-generation, lower-income, and marginalized students of color who disproportionately face food and housing insecurity, financial constraints, and additional obstacles navigating college life [9, 13].

Understanding unique barriers faced by underserved students in accessing, affording, and choosing healthy foods is crucial to promoting equitable campus environments that foster instead of hinder health, well-being, and success. HBCUs play a pivotal role in higher education, fostering the intellectual and cultural growth of a diverse student population. Despite their significance, HBCUs face unique challenges, and one such challenge is the promotion of healthy eating habits among their students [10]. However, research on individual and environmental determinants of dietary behaviors among racially diverse college populations has heavily emphasized predominantly white institutions, with few studies focused on minority-serving institutions [14]. There are also factors that can enable healthy eating among college students and create an environment that supports their nutritional needs. Perspectives from students and key stakeholders are needed to elucidate multi-level barriers and potential solutions specific to HBCU contexts toward reducing nutrition-related disparities. Consistent with social ecological frameworks, individual knowledge and motivations, social norms and supports, living conditions, institutional policies, and accessibility of healthy options within campus food environments interact to shape behaviors [15, 16]. These intricate relationships are best captured by qualitative investigations that value student voice and lived experience above reductionist surveys alone. Policy and customized, culturally appropriate initiatives that address structural gaps in campus resources, programs, and capacity to support diverse students in eating well can be informed by such observations. This helps them succeed academically and may lessen the burden of increasing chronic diseases that they are disproportionately affected by in later life [9].

This study therefore applies a qualitative approach to identify barriers and enablers to healthy eating experienced specifically among racially diverse students at an HBCU located in Texas. This study is particularly relevant as it focuses on an HBCU located in a food desert in Texas and made up of about 83.9% Blacks or African Americans. A food desert is an area with limited access to affordable and nutritious food, which exacerbates the challenges students face in maintaining healthy eating habits. Limited access to fresh produce and healthy food options forces students to rely on fast food and convenience stores, which typically offer less nutritious options. This environmental factor, combined with socioeconomic factors, cultural preferences, and the historical context of HBCUs, makes it more difficult for these students to adopt and maintain healthy dietary habits [12].

The primary objective of this study was to identify and analyze the barriers that impede students in the HBCU from adopting and maintaining healthy eating habits using the social ecological model (SEM). Additionally, the study aimed to garner potential solutions and interventions to overcome these barriers, promoting a culture of wellness within the HBCU community. Given the growing concern about the health of college-aged populations, this study contributes to the broader field of public health knowledge. Insights gained from HBCU-specific research can inform evidence-based interventions applicable to similar institutions, enriching the understanding of health dynamics among diverse student groups. Understanding the barriers to healthy eating using the SEM model can inform the creation of policies that support and promote a culture of wellness within HBCUs, fostering a healthier environment for their students.

## Method

### Study population and design

The World Health Organization’s social ecological model (SEM) offers a comprehensive framework for comprehending the diverse factors influencing an individual’s health and well-being. These factors encompass personal beliefs, interpersonal relationships, community policies, and environmental structures. The model, consisting of four interrelated components, acknowledges the intricate interplay of influences on health, particularly those related to social determinants, fostering lasting health promotion efforts among health professionals [17]. This model, organized into four interconnected components, recognizes the diverse factors shaping health outcomes. At its core lies the individual/interpersonal level, focusing on factors shaping an individual’s health and behavior, such as personal beliefs, attitudes, and unique characteristics. Moving outward, the interpersonal level encompasses the dynamics of relationships among individuals, including interactions within families, friendships, and broader social networks. The systems/community level addresses the healthcare delivery systems and broader infrastructure that can either support or impede health-promoting behaviors, spanning institutions like schools, workplaces, and neighborhoods. Lastly, the outermost layer is the policy level, encompassing local, state, and federal rules, policies, and laws regulating access to services, availability, legal rights, and protections [18].

**Fig. 1:**
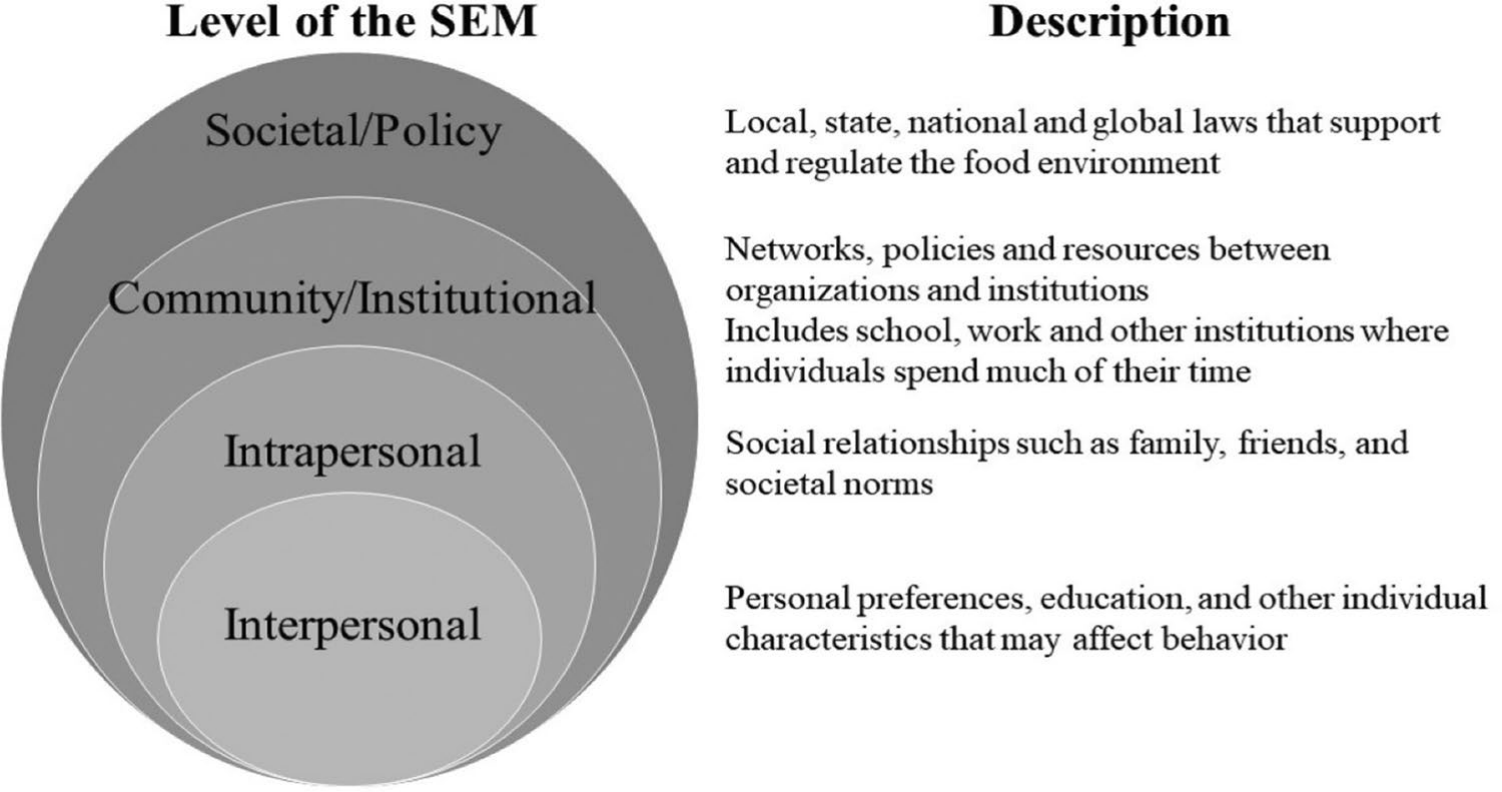
Social ecological model (SEM) levels [18, 19]

A qualitative approach comprising two sample groups was used to assess and obtain an understanding of the obstacles and possible solutions to healthy eating among the students of an HBCU located in a food desert at Texas. Key informant interviews (KIIs) were held with purposively selected significant stakeholders who had direct dealings with the food security initiatives on campus such as employees of the food pantry and health services, extension agents, the city community leaders, the cafeteria staff, students and staff of the student-led garden, the meal sharing program, and student government. E-mail messages were sent to stakeholders to inform them about the study and request their available times for an interview. Focus group discussions (FGDs) were also held among students. As part of their informed consent, participants got information about the study and all participants provided written consent. The research was authorized by the University Institutional Review Board. For the FGDs, participants were recruited via flyers that were distributed across the University facilities, and via email using a college student database. In the advertisement sheet, a link to an online survey was provided to facilitate recruitment and to give subjects the essential background to the study. Students who met the inclusion criteria were contacted via text message and email to provide their availability for the FGD sessions and only the eight students who responded participated in the FGDs. The discussion sessions were held both in-person and via zoom depending on the preference of the participants. Eligible participants included students who > / = 18 years of age, are food insecure, and/or have a diet-related chronic disease such as obesity, diabetes/prediabetes, or cardiovascular disease as these groups of people are impaired by unhealthy eating (Fig. 1).

### Data Collection and Analysis

Fifteen KIIs lasting between 20 and 40 min each and two 60-min FGDs with four students each were conducted. The discussion and interview sessions were audio-recorded and notes were taken by a research observer. Recordings were transcribed, and identifiers were redacted. The structured open-ended questions used in the FGDs and interview guides were provided by the principal investigator of the research team. For the KIIs and FGDs, questions were asked about the participants’ perception of the campus food environment and their experience with the different campus food security initiatives, and the students were further asked about their experience with healthy eating as students. The interviewers recorded the keywords and phrases used by the respondents and occasionally repeated the responses to confirm that they understood and interpreted what they had said. This allowed them to ensure that the data being collected was reliable.

The interviews were transcribed into Microsoft Word, anonymized, reviewed for accuracy, and then uploaded into QSR NVivo 12 Pro (QSR International Pty Ltd. Release 1.0, 2020). Thematic analysis was the method used. The information was coded and examined to identify emerging trends by two members of the research team. Where nodes included similar statements, they were merged. Statements were coded to themes based on concepts conveyed. Text-search queries helped find examples of where a respondent had addressed a theme in another context, and these remarks were parallel-coded [20].

## Results

Out of the 15 participants for the KII, there were 6 (40.0%) females and 9 (60%) males, comprising 4 (36.4%) students and 11 (63.6%) members of staff who were key stakeholders of resources on campus put in place to enhance healthy eating. Their years of experience with the phenomenon under investigation ranged from 5 to 30 years. There was a poor response to the call for FGDs hence only eight students participated in the FGDs. They were aged between 19 and 65 years old comprising 4 males and 4 females. Three (37.5%) of them were graduate students, 2 seniors, 2 juniors, and 1 sophomore. Seven (87.5%) resided off campus.

Analysis of the interviews and FGDs identified the challenging themes limiting students’ capability for nutritious diets categorized into three SEM sublevels. Ten subthemes emerged; the themes and their definitions are presented in Table 1. Four of the themes considered hindrances fell within the individual/personal level of SEM, four were within the community/institutional level, while two were classified at the policy/societal level. Nine dominant opportunity areas/solutions also emerged majorly falling within the institutional/community sublevel of SEM as shown in Table 2. Basic information of the participants giving the quotes is given using codes such as FGD_F for a female focus group participant and KII_M for a male key informant participant.

**Table 1:** Hindrances to healthy eating and SEM levels

Individual/personal level	
Gap in nutrition knowledge—This theme examines the discrepancies and deficiencies in individuals’ understanding of nutrition-related information, which can impact dietary choices and overall health	‘‘So the main thing diet wise, we do have a few students that I guess are attempting to change their diets. And we have had a few problems with students that try to avoid meeting their diet and not doing a proper vegetarian diet. And there is that the most of the dietary deficiencies we see are in individuals that try to restrict their diet and don’t do it properly.’’—KII_M “They’re not familiar with different types of fruits and vegetables. They just know what they know, and they stick to what they know”—KII_F
Not wanting to try new foods—This theme examines the reluctance or refusal to try unfamiliar foods, which can limit dietary variety and nutritional intake	‘‘It’s hard to get students to really try something new, especially in this day and age when most of them are used to McDonald’s and things like that, which is really unhealthy’’—KII_F ‘‘People are of habit and you and I grew up being raised on a certain type of food. It’s hard to change from what you’ve been doing’’—KII_F
Lack of cooking skills—This theme explores the insufficient ability or confidence to prepare meals from scratch, which can affect dietary habits and overall nutrition	“Maybe even offering cooking classes like I know that I have avoided my kitchen like plague. Like I have cooked in it maybe three times, like actual meals in there.”—FGD_F “A lot of these students, one, they don’t have the method to prepare those food. They don’t have stove or do utensils or whatever it is required. And so some of them just don’t have the knowledge. So you can give them a box of healthy food all you want. But if they do not know how to prepare it and how to put them together, it will stay in a corner and rot.”—KII_F
Difficulty balancing health with competing priorities—This theme explores the challenges the students face in maintaining a healthy lifestyle while managing various other demands and responsibilities in their lives	“For the average student or student leader or just engaged student who has so many things on their plate, you know, you lose attendance, you lose points if you don’t come to class all the time. Right? So sometimes you may want to go to breakfast and get some food, but you’re going to end up with the C in class if you miss too many class periods trying to eat breakfast. And then if you don’t wake up early enough to eat breakfast and you’re going to sacrifice eating, you may not be able to eat into the lunch or dinner and you may end up in a routine that you didn’t even expect to be in just because of what your class schedule is or what the rigor is or what the expectations of your professors are. So, trying to make sure that you can still circle back and prioritize yourself and your health is very important. I think, you know, some students do struggle with and those are very valid struggles’’—KII_M
Community/institutional level	
Insufficient food variety on campus—This theme examines the limited range of food options available on campus, which can affect students’ dietary diversity and nutritional intake	“I heard the stories of the students that went to the person who’s in charge of the cafeteria said, I’m a vegetarian. I paid for X number of meals per semester. What meals do I get that are vegetarian? And they were told, oh, we have mashed potatoes, we have green peas and we have salads also, that’s not variety.” “I remember I think this was last semester. I was on my way there and this girl was yelling, the vegetarians are hungry and the vegans are starving. You know, all the lack of diversity in the diet.”—FGD_M “I will say that the menus are pretty much the same, whether those are the burgers, the fries, the weekly or biweekly rotation of the main meals, the international meals, the pizza, that’s pretty much all of the same.”—KII_M
Unconducive cooking environment—This theme examines the various factors in the cooking environment that hinder the students from preparing meals effectively, which can negatively impact dietary habits and nutrition	“I think the biggest disadvantages for freshmen, they are pretty much entirely reliant on the MSC because they don’t have a full kitchen. If they’re living in the university college, they have a mini fridge and a microwave, and the mini fridge can only fit so many items for two people. And you know, most healthy foods aren’t going to be microwavable.”—FGD_F
Most extension programs are done outside the campus—This theme examines the tendency for extension programs, such as educational workshops, training sessions, and community outreach initiatives, to be conducted off-campus, which can limit student participation and engagement	“I’m amazed by the fact that we have an extension program here. But that program is not student focused. Students are adults and individuals that are in learning mode. So, I don’t really understand why they don’t turn part of the program back to the campus with a target audience here. There’s almost 9000 individuals that potentially can use information based on healthy eating and living habits as opposed to you focus back out to the large community.”—KII_M “We work in the community with most of our Title one schools. We’re housed here on campus, but we focus on the community. And it’s kind of hard to get students here as well because they’re taking their classes.”—KII_F
Difficulty accessing grocery stores—This theme explores the challenges students face in reaching grocery stores, which can limit their ability to purchase fresh, nutritious foods and impact their overall dietary habits	“A lot of international students struggle with transportation on this campus. And because they struggle with transportation, they also struggle with food access.”—FGD_M “I don’t have a car so my resource is going to be skewed. I don’t want to eat anything that’s not really sustainable. Like if I go to a grocery store one time a month, I’m not going to get something that I’m going to run out of in a week because I won’t be able to get it for three more weeks”—FGD_F “Even though we have a Brookshire brothers, it’s 15 min by car for sure. But it takes up to three hours walking, right? It’s hard to reach food physically.”—FGD_F
Policy level	
Difficulty accessing federal food assistance resources—This theme explores the challenges students face in accessing federal food assistance programs, which can hinder their ability to secure adequate nutrition and food security	“I will also say that college students have a difficult time accessing food stamps because it’s something weird. There’s like the expectation that if you’re under the age of 24 or 26, you’re still a dependent and they expect your family to contribute to your food”—FGD_F
High cost of healthy options—This theme examines the financial barriers students face in accessing healthy food options, which can impact their ability to maintain a balanced and nutritious diet	“It’s so much more expensive to eat healthy. And so, like change up your diet the healthy way, like they have grocery marts, like all the different spots and like you can get healthy stuff, but it’s way out of your budget.”—FGD_F

**Table 2:** Proposed solutions to hindrances

Community/institutional level	
Conduct cooking demonstrations—This theme explores the practice of providing live cooking demonstrations to educate students on how to prepare nutritious meals, improve their cooking skills, and promote healthy eating habits	“To teach them how to use it and then, you know, making it pretty quick and easy, I think roasting. I think just showing them what they can do with what they have is probably the easiest thing.”—KII_F
Improve gardening skills—This theme focuses on enhancing students’ ability to grow their own food by teaching practical gardening techniques and knowledge, which can contribute to better nutrition and food security	“it’s giving an opportunity to a lot of times getting students to try different things and even different parts of the things that we eat.”—KII_F “like giving people the tools to be able to grow their own underscores the empowerment aspect of gardening.’’—KII_M
Include nutrition and health education in classroom curriculum—This theme explores the integration of nutrition and health education into the classroom curriculum to promote students’ knowledge and practices related to healthy eating, physical activity, and overall well-being	“Why don’t we give them that important part of education, which is healthy living, which goes along with healthy eating.”—KII_F “When people are educated as to food and how it impacts the bodies and the body and the relationship between food and some diseases, then they might be more inclined to make different food choices. So, education is a key to getting past most of these food barriers.”—KII_F “Do nutrition classes and the question is whether you could make it a required freshman seminar, because you will have trouble with students volunteering to attend. And so I think if it gets done for credit and maybe a requirement, I think it would be beneficial and I think it probably could be incorporated within their required health class.”—KII_F
Increase food variety—This theme explores the importance of expanding the range of food options available in various settings, such as cafeterias, dining halls, and food programs, to enhance dietary diversity and meet the nutritional needs and preferences of the students	“I would just rather them have more food options and educate them about the food options. Before you didn’t have this. And now you have this”—KII_M “Not just an increase in variety, but also an increase in flavor. It’s just the ability for students to still be healthy and have the proper nutrients that they need and things that still taste good that they want to eat.”—KII_M
More flexibility in cafeteria hours—This theme examines the need for extended and more flexible cafeteria operating hours to better accommodate the diverse schedules of students, thereby improving access to nutritious meals throughout the day	“I see the issue with the way that the cafeteria operates in that students in many instances miss the noon meal because they have classes that go across the noon hour and they may cross 1:00 pm. And I think the cafeteria closes at two. So, if they get close at 1:50, they’ve got just 10 min to get to the cafeteria. And it may not actually be easy to do. So, I think students do miss the noon meal and then they’ll and even some miss the evening meal.”—KII_M “Expansion of the MSC hours for each meal period, whether that’s breakfast going to about 11am and lunch being from the goal 12 at 3 p.m. and the dinner from 4 to 8. That way students who have class get out of those times have a little bit more availability to make sure that they can eat during the day.”—FGD_F
Make more fruits and healthy choices available on campus—This theme focuses on increasing the availability of fruits and other healthy food options in various campus dining facilities to promote better nutrition and support healthy eating habits among students	“Advocate for an actual grocery store; we don’t have one and that is pretty, pretty important.”—FGD_F “Honestly, like, if they had, like, smoothies, like a smoothie bar, that would be great, or a vending machine with healthier options.”—FGD_M “I wish they would have like a carrot, cucumber, or celery, available for us not as a topping. Okay. And then fruit that’s not fruit salad in syrup because that’s like the fruit they have. If they have like, actual oranges, apples and bananas that are in the cellophane or fruit salad that’s not in the syrup.—FGD_F
Organize enticing nutrition education programs—This theme involves the creation and implementation of engaging and attractive nutrition education programs on campus to enhance students’ knowledge, skills, and motivation toward healthier eating habits	“The better way to get them on board with eating nutritiously is having another set of students that will show them how to prepare it. OK, that it’s not very time consuming. I think that the best way to get to a student is to have a student that speaks their language. And so we have to find deck with students, whether it’s a student club or a few individuals that we trained to talk about that”—KII_F “Try to just have something there that they’re going to want and just make things fun, make them enjoyable, they love music. They love being engaged, they love, just feeling an affectious spirit if you’re not feeling, you just lecture. Yeah, the audience is dead.”—KII_F “Like with older adults, even with children, you have to have some type of incentive. If there’s no incentive, they’re not coming to the program. They want something for you to be able to even just give them this education a lot of times it’s not enough. You may have one or two that are really there for the education, and that’s enough for them. But the majority, the vast majority, they want something. Or do you have shirts to give out? Do you have a pen to give out? Do you have a coupon to give out? Is there going to be food there.”—KII_F
Provide products not just produce—This theme focuses on expanding the availability of food products, including packaged and prepared items, that emphasize nutrition and health, alongside fresh produce, to offer a broader range of healthy food choices on campus	“Grow and provide different products and sell certain products even if we are in agriculture and in the human sciences, which is like home EC, we can be taking those products to make other value added products, do research, feed people, not just fresh fruits and produce or meat. But like you know cookies and chips” “It’s great to give them the food, but they’ll be even better to make those products. If you were a sophomore to a senior, and when you stay in the apartment you could cook. You know, if you’re a freshman, you can’t.”—KII_M
Provide transportation to grocery stores—This theme focuses on addressing transportation barriers by offering reliable and accessible transportation options for students to access grocery stores and markets, thereby improving their ability to purchase nutritious food items	“Transportation and maybe assistance with applying for food stamps. If something like that could be here on campus, that would help. Just to see if you’re eligible or have someone who’s trained to help you go through the application.”—FGD_F “Provide robust service on campus that actually has a schedule where they’ll take students from campus to the grocery store back”—FGD_F

## Discussion

The result of this qualitative study identified key enablers and hindrances to healthy eating faced by college students in a HBCU located in Texas. The themes were identified through semi-structured interviews with key informants and FGDs using the SEM as a conceptual framework. The challenging factors ranged from knowledge and accessibility limitations to competing priorities and unfamiliarity with nutritious alternatives. This study uncovered intersections between individual competencies, environmental defaults, and systemic policies impacting students’ dietary patterns. The findings align with and build upon previous research on barriers to healthy nutrition in the college demography where several hindrances emerged from the study, restricting students’ ability to make optimal dietary choices [21, 22]. Financial constraints were a major obstacle, preventing students from affording healthier specialty items. A systematic review by Matias et al. [6] similarly found college students view the expense as a significant barrier, gravitating toward cheaper fast food. The relatively high cost of healthy options is considered a policy-level issue that the government needs to better address; this has posed consistent obstacles, as college budgets often strain to cover basic needs [23]. This emphasizes systemic affordability and food security challenges students face, supporting Payne-Sturges et al.’s [9] insights into lacking nutritional equity on campuses. Students at HBCUs often experience more significant financial challenges compared to those at PWIs, limiting their access to healthy food options. Earlier studies [24, 25] found that food insecurity is a prevalent issue among college students, particularly at institutions with a higher proportion of low-income students which significantly hinders their ability to maintain healthy eating habits. Among the community/institutional-level barriers identified is the issue of difficulty with physical access to healthy food options. Many lacked physical access/transportation to full-service grocers, thereby relying on limited, repetitive campus options, this aligns with Nelson et al. [26] identification of environmental factors like minimal supermarket access as key impediments. Stress and time scarcity also hindered healthy eating, as found previously by Choi [27] that academic pressure negatively impacts diet quality among first-year students, also packed scheduling demands and inflexible dining operating hours afforded little time for intentional nutrition. This reinforces a previous study by LaFountaine et al. [28] linking high students’ workloads with food insecurity risks. The longstanding issue of poor nutrition understanding among youth continued in the college setting, aligning with Hilger-Kolb and Diehl’s [29] findings where students struggled to apply nutrition principles unsuited to their lifestyle constraints. Students tended to stick to familiar foods from their upbringing as found in a previous study [30].

Conversely, several enablers were identified to promote healthy eating habits among students some similar to findings from previous studies. Weaving food literacy and lifestyle behavior change concepts into coursework and campus programming sustains education beyond one-off events while aligning health with academic success [31]. Leveraging influential student culture leaders to serve as nutrition ambassadors conducting outreach provides relatable messengers to model attitudes and behaviors [32]. Enhancing affordable transportation assistance to proximal stores combined with subsidized fresh food or prepared meal delivery routing to campus removes locational availability barriers for carless students who cannot easily obtain diverse ingredients for healthful cooking [33]. Increasing variety and encouraging new foods could significantly impact choices [22]. PWIs typically offer a wider variety of healthy food options on campus, including salad bars, vegetarian and vegan options, and other nutritious meals. The availability of these options is highlighted by Laska et al. [34]. Identified enablers offer promising conduits for positively influencing trajectories beyond the campus environment [35].

The hindrances and enablers highlight interdependencies between individuals and systems in shaping dietary behaviors. These findings indicate students face systemic hindrances blocking the capability to consume balanced diets, despite individual motivations [36]. The hindrances found reflect broader systemic barriers facing students seeking healthy options, including time poverty, financial limitations, unfamiliarity with nutrition, and environmental/policy restrictions on campus and in surrounding communities. As Horacek et al. [12] found, students hold positive nutrition attitudes but struggle enacting them amid barriers. The study makes clear that health promotion requires reducing hindrances while incorporating enablers at individual and institutional levels. Campus communities must make the healthy choice the easy choice. The enablers underscore practical, micro-level solutions institutions can implement, like variety, affordability, convenience, and peer demonstration. This interplay of macro-level hindrances and micro-level enablers mirrors the socioecological frameworks put forth by Stok et al. [34]. As the study shows, multidimensional factors intersect, demanding multitiered approaches spanning individual skill building to systemic change. Progress requires multi-sector coordination addressing root access inequalities, while uplifting student voice in decisions impacting their health [14]. This study contributes needed qualitative insights to guide initiatives tackling poor dietary habits contributing to concerning health trends among college populations.

This study has some limitations that should be taken into account when interpreting the findings. Conducted at an HBCU located in a food desert, most participants in our qualitative study lived off-campus. The small sample size of the focus group discussions may limit the generalizability of our findings. Despite this, the study’s insights could inform policy development at both institutional and governmental levels in similar contexts. The study on barriers and solutions to healthy eating among students in HBCUs holds significance not only for the well-being of the student population but also for advancing our understanding of health disparities, contributing to public health knowledge, and fostering a culture of wellness within the HBCU community. The outcomes of this research have the potential to drive positive change and serve as a model for promoting health and well-being in diverse educational settings.

## Data Availability

The datasets used and/or analyzed during the current study are available from the corresponding author on reasonable request.
